# Analytical Evaluation of an NGS Testing Method for Routine Molecular Diagnostics on Melanoma Formalin-Fixed, Paraffin-Embedded Tumor-Derived DNA

**DOI:** 10.3390/diagnostics9030117

**Published:** 2019-09-12

**Authors:** Irene Mancini, Lisa Simi, Francesca Salvianti, Francesca Castiglione, Gemma Sonnati, Pamela Pinzani

**Affiliations:** 1 Department of Experimental and Clinical Biomedical Sciences “Mario Serio”, University of Florence, 50139 Florence, Italy; irene.mancini@unifi.it (I.M.); francesca.salvianti@unifi.it (F.S.); sonnatigemma@gmail.com (G.S.); 2 Molecular and Clinical Biochemistry Laboratory, Careggi University Hospital, 50139 Florence, Italy; lisa.simi@unifi.it; 3 Histopathology and Molecular Diagnostics, Careggi University Hospital, 50139 Florence, Italy; francesca.castiglione@gmail.com

**Keywords:** melanoma, next generation sequencing, BRAF V-raf murine sarcoma viral oncogene homolog B, somatic mutation, sequenom MassARRAY system

## Abstract

Next Generation Sequencing (NGS) is a promising tool for the improvement of tumor molecular profiling in view of the identification of a personalized treatment in oncologic patients. To verify the potentiality of a targeted NGS (Ion AmpliSeq™ Cancer Hotspot Panel v2), selected melanoma samples (*n* = 21) were retrospectively analyzed on S5 platform in order to compare NGS performance with the conventional techniques adopted in our routine clinical setting (Sequenom MassARRAY system, Sanger sequencing, allele-specific real-time PCR). The capability in the identification of rare and low-frequency mutations in the main genes involved in melanoma (*BRAF* and *NRAS* genes) was verified and integrated with the results deriving from other oncogenes and tumor suppressor genes. The analytical evaluation was carried out by the analysis of DNA derived from control cell lines and FFPE (Formalin-Fixed, Paraffin-Embedded) samples to verify that the achieved resolution of uncommon mutations and low-frequency variants was suitable to meet the technical and clinical requests. Our results demonstrate that the amplicon-based NGS approach can reach the sensitivity proper of the allele-specific assays together with the high specificity of a sequencing method. An overall concordance among the tested methods was observed in the identification of classical and uncommon mutations. The assessment of the quality parameters and the comparison with the orthogonal methods suggest that the NGS method could be implemented in the clinical setting for melanoma molecular characterization.

## 1. Introduction

In recent years, the most important revolution in solid tumor management has been represented by the development of targeted therapies and the adoption of molecular criteria for precision diagnosis [1]. The capability to perform molecular profiles of cancers has greatly contributed to the identification and the enlargement of approved anti-cancer drugs as well as to refining the prediction of response in subgroups of patients harboring specific alterations. Since the identification of somatic mutations plays a central role in the era of personalized and precision medicine, the development of new molecular methods is a challenge in the research field but also an important improvement for the diagnostic laboratories.

Common methods applied to the molecular screening of tumor samples such as real-time polymerase chain reaction (qPCR) and Sanger sequencing are able to perform a single-mutation or single-exon test for each gene. Technological progress in the throughput has been reached by the introduction of different approaches for the parallel investigation of several hot-spot mutations in different genes. In the context of somatic mutations detection, these systems are mainly represented by the array platforms, combined with the chemistry used in qPCR [2] and the analysis of multiple extension fragments by mass-spectrometry [3,4,5].

As it is well known, the principal issue related to the analysis of somatic alterations is the sensitivity of the adopted method in terms of limit of detection (LOD) of the fraction of mutated allele. LOD represents the major weakness connected to the use of direct sequencing that, nevertheless, is still considered a reference method due to its high specificity. Generally, tumor sample genotyping derived from the use of multiple technological approaches guarantees a qualified response in terms of specificity, coverage of different variants, and sensitivity [6]. 

In this context, the emerging application of NGS technologies represents an appropriate alternative able to merge the high specificity and the coverage of a sequencing method with the sensitivity proper of the allele-specific molecular assays like qPCR.

More recently, the increased number of genetic alterations clinically significant for the oncologists allowed to focus on other critical topics extremely relevant in the context of tumor specimens. In particular, the amount of nucleic acid required for the setting of a wide panel of mutations is becoming a crucial aspect to be considered for the feasibility of a multi-marker evaluation on small biological samples [2]. Nowadays, the NGS methods based on the sequencing of amplicon-libraries have solved this problem limiting the amount of starting material necessary to perform the analysis [7]. By this approach, large genomic regions can easily be targeted through the amplification of small quantity of the nucleic acid with high multiplexed pools of primers, avoiding sample consumption [8,9].

In melanoma, targeted therapies are optimized for patients bearing activating mutations in the *BRAF* and *KIT* oncogenes [10,11,12]. Although these are particularly useful, other alterations in driver genes, such as *NRAS* or *GNAQ* and *GNA11* [13,14,15], could become new potential targets of antagonist drugs or fundamental decision markers of a treatment strategy and prognosis [16].

The activating p.Val600Glu mutation in *BRAF* is considered the main important alteration detectable in melanoma samples due to the high occurrence in up to 95% of *BRAF*-mutant melanomas [17] and to the improvement of survival in response to BRAF-inhibitor treatments in patients affected by metastatic melanoma harboring this mutation [18].

However, other non-classical BRAF mutations are reported with lower incidence in melanoma samples [19,20,21]. Depending on their final effect on the protein, BRAF-mutated tumors with a rare variant could be similarly treated with BRAF-inhibitors, with a relevant impact on the disease-free and overall survival of these patients.

For this reason, the use of technological platforms, able to detect with high sensitivity and specificity all actionable mutations, seems to be the best choice to extend the therapeutic option to patients currently excluded from targeted therapies or clinical trials.

Application of massive parallel sequencing by NGS has prompted a widespread use of the information related to tumor genotype (as the concurrent mutations in the same sample) for a deeper definition of the potential association with the clinical response and with the resistance mechanisms [21].

Similarly, another important unresolved issue to date is the variability in the molecular profiles among synchronous or asynchronous melanoma metastases [22] and that observed in the primary tumor of the same patient [23,24]. The newly developed NGS approaches through the analysis of wide panels of genes allow a careful definition of the tumor’s clonal evolution by detecting the variation in the number, the type, and the rate of mutations among multiple samples from one individual patient. 

This study investigates the potentiality of a targeted NGS approach (Ion AmpliSeq™ Cancer Hotspot Panel v2) for the assessment, in a single assay, of hot spot mutations in 50 oncogenes and tumor suppressor genes involved in cancer. The evaluation of the analytical performance of the adopted NGS system was carried out by the analysis of DNA derived from cell lines (of known genotype, used as control samples) and FFPE samples to verify that the detection of non-common mutations and low-frequency variants were adequate to meet the technical and clinical requirements for the principal genes investigated in melanoma. Moreover, archived melanoma samples were retrospectively chosen and analyzed on the IonS5 platform (Thermo Fisher Scientific) to compare the results with those obtained by the conventional techniques adopted in our routine clinical setting (Sanger sequencing, Sequenom MassARRAY system, competitive allele-specific real-time PCR) and evaluate the deriving improvement in the molecular profiling of tumor samples.

## 2. Materials and Methods

### 2.1. Samples

For therapeutic purpose, the *BRAF* and *NRAS* mutational status of 136 melanoma samples was investigated in two years by conventional techniques adopted in the routine clinical setting. 

Among those, twenty-one collected DNA samples were retrospectively chosen, based on *BRAF* genotype, to evaluate the performance of an NGS application for the molecular analysis of melanoma samples. The series included samples harboring classical and rarer *BRAF* Val600 variants at different percentage of mutated allele, non-Val600 variants and wild-type samples for the presence of a mutation in exon 15.

Genomic DNA was isolated from formalin-fixed paraffin-embedded (FFPE) tissues samples using the QIAmp DNA FFPE Tissue Kit (Qiagen, Hilden, Germany) following the manufacturer’s instructions after a proteinase K overnight digestion at 56 °C. Control DNA samples from cell lines were isolated using QIAamp DNA Mini Kit (Qiagen, Hilden, Germany).

DNA quantity was evaluated using NanoDrop ND-1000 Spectrophotometer (Thermo Scientific, Inc., NYSE:TMO) and Qubit™ dsDNA HS Assay Kit on Qubit 3.0 Fluorometer (Thermo Fisher Scientific, Carlsband, CA, USA).

The research protocol was approved by the institutional review board of the University of Florence. All the patients gave written informed consent at their own Division of Medical Oncology (in accordance with the local Institutional Ethical Committee) for testing of the requested markers and agreed to the research use of the tumor specimens.

### 2.2. BRAF Mutation Analysis from Melanoma Tissues by Conventional Methods

The *BRAF* genotype for melanoma samples was derived from the use of more than one technique to guarantee a qualified response, in terms of specificity and sensitivity.

In first line, all samples were submitted to a mass-spectrometry analysis by using the Sequenom MassARRAY system (Sequenom, San Diego, CA, USA) and Myriapods Colon Status Kit (Diatech Pharmacogenetics, Jesi, Italy). The panel consists of 59 assays multiplexed in 8 wells to detect the main colon and melanoma cancer-related mutations in *KRAS, BRAF, NRAS*, and *PIK3CA* genes [25].

Briefly, 10 ng of DNA, for a total of 80 ng, was added to each well and amplified and extended following the manufacturer’s protocols. PCR reactions were treated with shrimp alkaline phosphatase (SAP) to remove unincorporated nucleotides and primers. The purified products were submitted to a single base extension reaction (iPlex) by using primers that anneal immediately adjacent to the hot spot mutation base. A purification of salts was done by adding a cation-exchange resin after the primer extension. The final products were spotted on a SpectroCHIP II arrays by MassARRAY Nanodispenser RS1000 and analyzed by MALDI-TOF in the MassARRAY Analyzer 4 Instrument. The spectral profiles were resolved by the application of the Typer Analyzer of Typer 4.0 software and elaborated through Dossier software developed by iGENETICS MYRIAPOD (BiMind Srl, Jesi, Italy).

For *BRAF* wild-type samples, a second analysis was performed by a pre-screening method (High Resolution Melting Analysis) followed by the Sanger sequencing of *BRAF* and/or *RAS* genes and, subsequently, a competitive allele-specific real-time PCR method in order to identify (i) the presence of a rare *BRAF* mutation out of the hotspot mutations interrogated and (ii) the presence of a low fraction of mutated p.Val600Glu allele.

The DNA samples were screened by High Resolution Melting Analysis (HRMA) in a RotorGene6000 system (Qiagen, Hilden, Germany), and the results were confirmed by sequencing as previously reported [26].

Finally, wild type samples were analyzed by castPCR (competitive allele-specific TaqMan PCR; Thermo Fisher Scientific, Inc., Waltham, MA, USA) technology by using the BRAF_476_mu (Assay ID: Hs00000111_mu) probes with the corresponding wild type allele assays BRAF_476_wt (Assay ID: Hs00000110_wt) and the gene reference assay BRAF_rf (Assay ID: Hs00000172_rf) in a StepOnePlus Real-Time PCR System (Thermo Fisher Scientific). Analysis was performed by using the Mutation Detector software v.2.0 (Thermo Fisher Scientific) to confirm the wild-type genotype or to evaluate the *BRAF* p.Val600Glu allele fraction.

### 2.3. Analysis by Amplicon-Based Next Generation Sequencing

#### 2.3.1. Library Preparation and Sequencing

NGS workflows and analysis were performed following manufacturer’s instructions using Ion AmpliSeq™ Cancer Hotspot Panel v2 (Thermo Fisher Scientific, Inc., Waltham, MA, USA) for DNA analysis.

Ten nanograms of DNA was used as template in library construction. The DNA panel, consisting in a single pool of 207 primer pairs, includes hot spots regions of 50 oncogenes and tumor suppressor genes, among which *BRAF, NRAS, KRAS*, and *PI3KCA* (also included in the Sequenom panel) and other genes reported to be mutated in melanoma samples (*KIT*, *CDKN2A*, *ERBB4*).

The target regions (mean length = 154 bp) were amplified by 20 cycles with an extension time of 4 min as suggested for tissue samples derived from FFPE specimens. Each library was identified by a unique IonXpress barcode. The DNA libraries were quantified by using the Ion Library TaqMan^®^ Quantitation Kit. After proper dilutions, ten DNA libraries were combined to obtain 100 µL of pooled libraries (8 µM) for emulsion PCR. Emulsion PCR was performed on Ion One Touch 2 Instrument with the Ion 520™ & Ion 530™ Kit–OT2 200 bp to prepare template-positive ISPs (Ion SphereParticles). The success of the reaction was verified with the Ion Sphere Quality Control kit AlexaFluor 488 and 647. The following enrichment of ISPs was performed on the Ion One Touch Enrichment System. Enriched positive-template ISPs samples were loaded in an Ion 520 chip (3–6 million reads/chip. Output per run: 0.6–1 Gb) and run on the Ion S5 Sequencing Systems to be sequenced for somatic mutations analysis.

#### 2.3.2. Calling and Filtering Variants, Annotation and Interpretation

The automated data analysis was performed with Torrent Suite version 5.2. The DNA raw reads were aligned back to the *hg19* (human reference genome) and results of coverage analysis verified for each amplicon. The variant calling and the annotation were carried out by Ion Reporter version 5.2. The alignments and the presence of filtered-in variants were visually confirmed with Integrative Genomics Viewer (IGV v2.3) or Golden Helix GenomeBrowse (v2.1.2).

Sequence variants were firstly annotated by using Ion Reporter whereas, in some instances, other tools (i.e., SIFT, Polyphen and Mutation tasting) and/or the literature were searched to verify the prediction on the pathogenicity of some unclassified variants.

### 2.4. Statistical Analysis

Statistical analysis (Pearson’s correlation coefficient and subsequent significance (2-tailed) and distribution of the coefficient of variation) was carried out using the IBM Corp. Released 2017. IBM SPSS Statistics for Windows, Version 25.0 (IBM Corp: Armonk, NY, USA) software package.

## 3. Results

### 3.1. Evaluation of the Precision of the NGS Assay and Bioinformatic Tool

The assay sensitivity was assessed by sequencing serially diluted mixed DNA samples from cell lines harboring known and distinct somatic mutations as reported in Table 1 (COSMIC cell line project: https://cancer.sanger.ac.uk/cell_lines).

Three dilution samples with a variable amount of allelic variants for each specific gene (MIX A, B and C) were prepared as reported in the Table 1 and used as reference. From each reconstituted mixed sample (A, B, C), two independent libraries (1 and 2 barcodes) were prepared and sequenced twice (I and II chips) in consecutive experiments (first and second runs).

**Figure 1:**
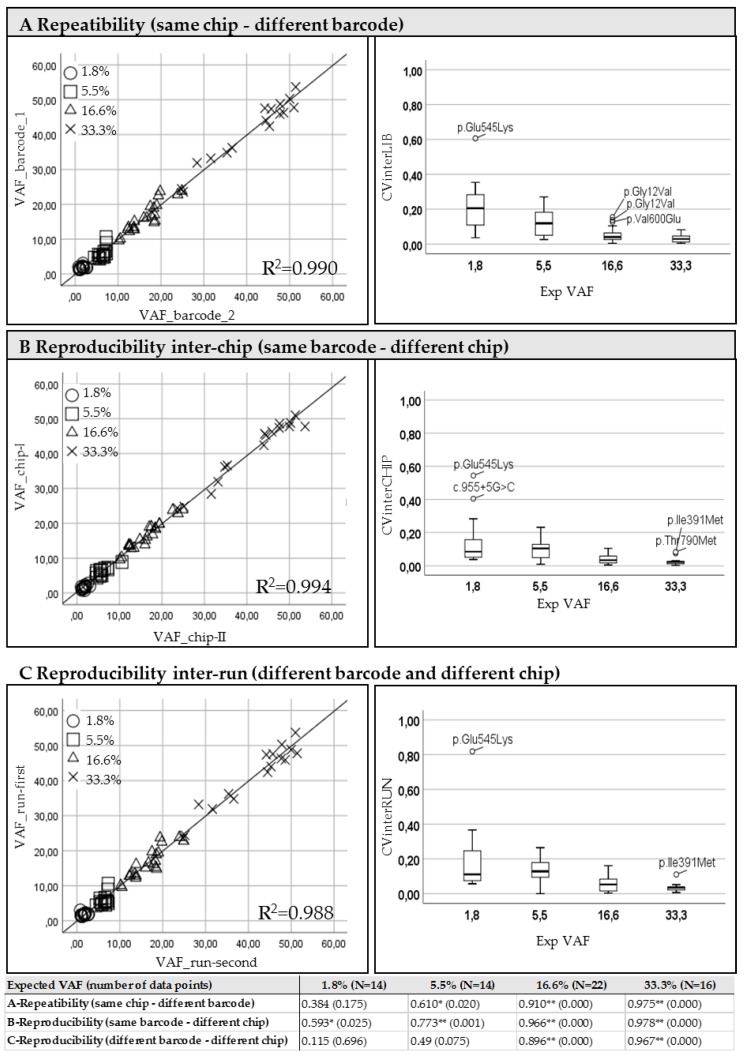
Repeatibility and Reproducibility of the NGS method. Diluted mixed DNA samples from cell lines were analyzed using different barcodes (**A**) or on different chips (**B**) or with different barcodes in two consecutive runs (**C**). Left panels: Each section reports the correlation between the returned VAFs in the specific evaluation experiment. In the scatter graphs, the Pearson’s correlations are illustrated grouping data using different symbols depending on the expected VAFs. Pearson’s coefficients for each expected VAFs are reported in the table at the bottom of the figure. Significant correlations are flagged with one star (*) or two stars (**) if the p-value is less than 0.05 and 0.001, respectively. Right panels: the box-plots represent the distribution of the coefficients of variation obtained for each experimental condition at different expected VAFs. Outliers are indicated by the specific mutation change.

The correlation of Variant Allele Frequencies (VAFs reported in the left panels of Figure 1) resulting from replicates was used in order to verify the inter-library repeatability using different barcodes (Figure 1A: intra-run, samples 1-I vs. 2-I and 1-II vs. 2-II) and the reproducibility inter-chip (Figure 1B: intra-library, samples: 1-I vs. 1-II and 2-I vs. 2-II) and inter-run (Figure 1C: inter-library, samples: 1-I vs. 2-II and 2-I vs. 1-II).

A high concordance was obtained for all mutations expected in the mixed DNA samples at least at 5.5% frequency of mutated allele (see Table 1), in particular when the same library was run in different chips (reproducibility inter-chip in panel B). A weaker correlation was observed between different libraries from the same sample loaded into the same chip (repeatability in panel A) especially for the variants present at the investigated dilution point of 1.8%.

As expected, the relative coefficient of variation (CV) of the frequencies obtained for all known mutations, resulting from both inter and intra-run analysis, evidenced an increase of the variance at the reduction of the expected percentage of mutated allele (Figure 1, right panels).

Moreover, for the investigated hot spots, we evaluated a common error-rate of 0.1%, which reached the 0.8% in case of homopolimeric regions (e.g., in the genomic position chr3:178936082 in hg19 interested by *PIK3CA* codon 545) arising from the number of reads containing a false-call at the level of known sequence variants.

Since at the highest point of dilution (with expected of VAF 1.8%) an increased variability was observed, we predicted a cut-off of 5% sufficiently reliable, even if the identification of lower frequency mutations was not excluded.

To assess the limit of detection for variant calling by the Ion Reporter Software, the mixed DNA samples were analyzed by the adopted workflow with default parameters established for somatic mutation analysis. All expected mutations of cell MIX A, B, and C, containing at least 5% of mutated allele, were identified in the duplicate of the libraries in both runs (variant call *p*-value = 0.00001) (data not shown).

Differently, the sequence variants expected at 1.8% were not always detected in the replicates of the cell MIX C. Despite all mutations could be identified by visual check (even if in a small number of reads), the variant caller software was not reproducible in the detection of the variants, producing different results for the same hot spot among the replicates as reported in Table 2: mutated with weaker statistical significance (variants called with *p*-value > 0.005), mutation “no-call” for poor quality or not variant detected (REF: wild type). Therefore, the uneven behavior of variant caller observed for low-frequency variants confirmed that it is unfeasible to define a unique limit of detection for all variants in a panel and that reproducible results could be obtained for all mutations with an expected VAF around 5%.

To mimic the detection of low-frequency mutations in a DNA sample derived from an FFPE specimen, the repeatability and the reproducibility were similarly verified on a DNA obtained by mixing two samples (MEL3 and MEL5) in a ratio of 2:1. The expected variant frequencies were 7.9% for *BRAF,* 5.6% for *NRAS*, 4.8% for *KIT* and 3.7% for *ERBB4* calculated from the returned VAFs of unmixed samples by NGS. Similarly, a duplicate of the library was run in two consecutive independent sequencing experiments to assess the reproducibility of the analysis.

The concordance in variant calling between different runs of the same library was complete. Otherwise, the pairwise concordance (inter-libraries) from the duplicate failed for the variant at lowest allele frequency. The mutation in *ERBB4* p.Asn174Ser (expected at 3.7%) was not called in the second library of the mixed sample, but it was visible at the examination of BAM (Binary Alignment Map) file in a small number (lower than 100 with a mean coverage of 2864) of mutation-containing reads corresponding to the 3% (Data not shown).

In view of that evidence, the identification of low-frequency variants has been considered strongly affected by the amplification step required for the libraries’ construction, in addition to other possible factors as the sequencing context and the specific base substitution. Therefore, the limit of detection of 5% has been confirmed sufficiently reliable for the subsequent analysis in melanoma samples and, theoretically, able to detect a heterozygous mutation in a sample with 10% of tumor cells. As a rule, the visual inspection of raw data was established mandatory for pathogenic variants identified with a VAF < 5% before reporting the result as informative. Moreover, for wild-type genotypes in the most significant genes, the visual inspection of reads was done to prevent false negative results and to consider the usefulness of a complemental confirmatory test.

### 3.2. Coverage Analysis and Quality Parameters of FFPE Samples

All the libraries were successfully amplified and passed the minimal requirement (20 pM/L) for all the samples. The uniformity of coverage ranged from 87.6% to 100% among different DNA samples. The percentage and the amplicons with at least 500 × or 100 × coverage were calculated and verified to estimate the robustness of this panel for targeting DNAs extracted from FFPE samples and to exclude possible false negative results. Only in two samples the panel was covered less than 90% but without involving the main targets for melanoma as *BRAF* and *NRAS* genes. Quality control metrics are reported in Table S1 (supplementary data).

### 3.3. Concordance between NGS and Genotyping by Mass Spectrometry for Common Mutations in BRAF, NRAS, KRAS and PIK3CA

Firstly, a comparison between the NGS data and the results obtained from the Sequenom MassARRAY System (by Myriapods Colon Status Kit analysis) was performed to verify the variant calling accuracy and to endorse results for the most common hot spots in the genes *BRAF*, *NRAS*, *KRAS*, *PIK3CA*.

Seven most common *BRAF* mutations (supplemental Table S2) involving the codon 600 (3 classical p.Val600Glu, 1 p.Val600Glu complex, 1 p.Val600Lys, 1 p.Val600Arg, 1 p.Val600_Lys601delinsGlu), four variants in the *RAS* gene family (3 in *NRAS*; 1 in *KRAS*) and two in *PIK3CA* were correctly identified by NGS. Additionally, in two different samples, a coding variant introducing the amino acid change p.Val600Glu and p.Val600Arg was identified respectively at 3.62 and 4.9% of allele frequency (supplementary Table S3). The presence of these low-percentage mutations was previously evidenced only by the castPCR method used as confirmatory method on BRAF wild-type samples to increase the sensitivity of the screening test. The presence of the p.Val600Arg was probably identified through cross-reactivity process of the castPCR assay, despite its specific design for the p.Val600Glu variant. Finally, a further mutation in *NRAS* gene, missed by Sequenom analysis, was detected at 19.5% and confirmed by conventional direct sequencing in one case harboring a *BRAF* gene mutation at low frequency.

### 3.4. Detection of Rarer BRAF Mutations by NGS

The general capability of the NGS panel to detect rare *BRAF* mutations was also verified by melanoma samples analysis.

To assess the capability of the NGS approach in the detection of uncommon mutations, five samples positive for an unusual mutation in the *BRAF* exon 15 not involving codon 600 (p.Asn581Ser, two p.Asp594Asn, p.Gly596Val, p.Leu597Gln), previously identified only by the use of direct sequencing, have been tested (supplemental Table S4).

All mutations were correctly detected and called, displaying a perfect concordance of results between NGS and Sanger sequencing in rare sequence variants discovery. Among the five melanomas harboring a rare *BRAF* mutation, two samples with the *BRAF* p.Asp594Asn showed an additional mutation in the *NRAS* (p.Gly12Asp) or in the *KRAS* (p.Gln61His) gene, previously identified in routine analysis. Finally, among five samples known to be wild-type for *BRAF* and *NRAS* (supplemental Table S5), the NGS allowed the identification of two samples, belonging to the same patient, with a *BRAF* mutation in exon 11 (p.Gly466Arg) concurrently with the p.Gly12Asp in the *HRAS* gene.

A complete overview of the results obtained by NGS compared with data from the routinely applied methods is reported in Tables S2–S5 (supplementary data).

### 3.5. Detection of Additional Mutations by NGS in Melanoma Samples

**Figure 2:**
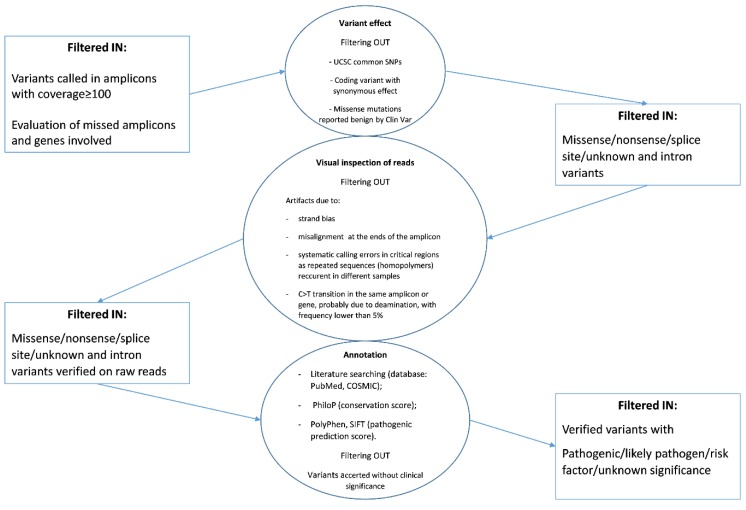
Flow chart of filter chain used for the prioritization of variants detected by NGS.

The sequence variants called were annotated and classified according to the workflow reported in Figure 2 starting from the coverage analysis of the amplicon regions.

Briefly, the majority of known variants were filtered out because they were reported as (i) common SNPs (checked in dbSNP), (ii) missense variants confirmed benign in ClinVar database, and (iii) variants with synonymous effect at the protein level. Subsequently, a visual inspection of annotated variants was performed by the Genome Browse to exclude technical errors. Finally, the pathogenic or presumed pathogenic variants and those “with unknown/uncertain significance” occurring in informative amplicons were prioritized and their hypothetical effect evaluated by bioinformatics tools and literature searching. All variants identified are reported in Figure S1 and samples are categorized in relation to the ascribed effect of the detected *BRAF* mutation on the protein activity.

## 4. Discussion

To increase precision in cancer diagnosis and its treatment options, a wide spectrum of reasons supports the implementation of NGS technologies in the clinical diagnostic setting. The number of targetable genes is steadily growing and, subsequently, an efficient system to simultaneously examine complex gene panels (instead of just one single target) to achieve a wider and accurate tumor molecular profiling is strongly encouraged. Actually, the breakthrough and adoption of new drugs is strictly connected to the acquisition of several genetic features during cancer development. In this context, the evaluation of multiple exons and of new entire genes is becoming almost a standard in diagnostic practice. For that reason, the availability of flexible, scalable, and easy-to-improve NGS technologies seems to provide the best support for the constant upgrading required by the clinical laboratories. For oncological purposes, several ready-to-use NGS panels are now commercially available, and many studies have already validated the use of NGS to screen multiple hotspot mutations in DNA samples derived from FFPE tissues [28,29,30,31,32].

The preset study reports our experience with NGS, performed by the Ion AmpliSeq Cancer Hotspot Panel v2 on the IonS5 platform, in comparison with the routinely used methods for melanoma molecular analysis.

Summarizing, we assessed the performance of the NGS assay by sequencing DNAs from cell lines and twenty-one well-characterized FFPE samples. The final aim was to verify the NGS capability in replacing our multi-modal approach, represented by a mass-spectrometry multi-target evaluation combined with confirmatory tests based on single-exon (sanger sequencing) or single-mutation (allele-specific qPCR) assays. The selection of samples was achieved to build a series of samples covering sufficient types of possible variants.

Our results confirm that a univocal limit of detection for all the NGS assays composing the panel cannot be easily defined due to the different variant types and to the sequence context influence. Therefore, we have considered more suitable to evaluate the general sensitivity by establishing the variant allele frequency at which the variant caller confidently detected all expected variants in our reference samples.

The analysis of DNA samples from cell lines with known level fraction of mutation shows an exponential increment of variability across replicates with the reduction of the allele frequency. As reported in previous studies [7,29,33,34], the analysis of reproducibility and repeatability allowed us to detect consistently different types of sequence mutations present in the sample at a frequency of 5%. However, depending on the quality of the reads and sequence context, we cannot exclude the possibility to identify a mutation at lower frequency. Thus, a threshold was not applied in order to maximize the sensitivity of the NGS assay. On the contrary, to prevent false-positive results, the visual inspection of all mutations identified by using the Ion Reporter Software was done, and some suspected technical artifacts were suspended pending confirmations. By this approach, two melanoma samples with a mutation at very low frequency in the *BRAF* gene, missed both by mass-spectrometry and Sanger sequencing, were correctly identified, confirming the high sensitivity of NGS method comparable to the previous screening by competitive allele-specific PCR.

Furthermore, we verified the analytical validity of the NGS results by sequencing those FFPE samples for which the genotype was well characterized for the presence of a mutation in the *BRAF* gene or the total absence of mutations in other routinely investigated genes. A high concordance between NGS and direct sequencing was observed in the identification of classical and uncommon mutations involving the exon 15 of *BRAF* and exons 12 and 13 of *RAS* genes. Variant caller software was able to annotate with high accuracy all sequencing variants, previously detected by Sanger sequencing, present in the samples at VAFs between 19.5% and 63.4%. Moreover, the NGS analysis allowed the correct classification of a false negative sample for a classical variant at codon 61 of *NRAS* gene undetectable by mass-spectrometry due to the low-quality spectra of the specific mutation-assay. No other discrepancies have been observed between the mutations investigated by the Sequenom panel and their re-evaluation by NGS.

Overall, our evaluation showed that this amplicon-based NGS method fulfils the main technical requirements for the molecular characterization of melanoma samples. In particular, we verified that comparable sensitivity and specificity for the detection of several types of somatic mutations could be easily achieved by the use of a unique method thanks to the implementation of NGS technology. Additionally, NGS analysis provides information on the variant frequency that cannot be obtained by the mass-spectrometry analysis or conventional direct sequencing. In the future, we expect that, as shown for anti-tyrosine kinase agents [35], the quantitative assessment of a specific sensitive-mutation may become an important parameter to predict the entity and the duration of the response to specific targeted therapies in melanoma patients.

Despite these advantages derived from the implementation of NGS, the translation of these technologies into clinical laboratories has proved the need for standardizing reference materials to verify the test performances among different laboratories and to compare results obtained from different platforms, gene panels, and bioinformatics pipelines.

Due to the complexity of several sequences to be tested and the high number of technical variables, from the matrix of the starting samples to the bioinformatics analysis, the choice of the best reference material could not be easy.

In particular, a stable, abundant, and well-characterized source of a cancer genome to mimic a real sample is quite impossible to obtain. Several synthetic DNAs or spike-in controls, developed to address specific genetic features, are now commercially available. Analogously, mixed DNAs from tumor-derived cell lines could provide a renewable sample easily accessible and cheaper for the validation phase in many laboratories [36]. Despite these control materials being particularly useful for the definition and monitoring of quality parameters over time, they may not possess a comparable behavior with a real genomic sample through all the different phases required in the analytical process [37]. On the contrary, archived genomic DNAs from tumor specimens could represent a perfect reference material commutable with real samples because they are able to address the precision of the detection in routine conditions, especially in case of rare mutations, but they could be limited in the amount and in the genetic characterization.

Therefore, we have chosen to evaluate the performance of the NGS panel by adopting a combination of laboratory-developed reference materials: first, the reliability was verified by the analysis of mixed DNAs from cell lines, commonly used as controls in our routine analysis; then a second assessment was performed by using FFPE-derived DNA samples extensively characterized by conventional molecular analysis.

To perform the full characterization required in our diagnostic setting, the entire process of the NGS resulted faster and required less DNA than would be necessary to perform multiple separate tests with conventional methods. These aspects are fundamental, especially in the context of melanoma, to minimize the number of the working days necessary to reach the results (around 5 days) and to avoid unnecessary precious sample-consumption for confirmatory tests.

By comparison, the mean cost of consumables for conventional methods, useful for the screening of a limited number of targets, and for a wide panel performed by NGS is comparable [38]. In the future, the NGS approach should become even more advantageous with the increase of investigated regions and the optimization of the number of pooled samples loadable into the same chip.

Moreover, the introduction of NGS could enable the detection of multiple regions of genes overcoming the persistent concern of the amount of available tissue in case of highly limited specimens. For example, two putative wild-type samples included in our study revealed the presence of a *BRAF* mutation in the exon 11. In these samples, a concomitant mutation in *HRAS* gene (p.Gly12Asp) was identified, confirming the association between inactivating *BRAF* mutations and the stimulation of the Raf-MEK-ERK pathway by other effectors, as a mutated RAS protein, in a molecular subtype of melanomas. The wide analysis performed by NGS can also provide additional genetic information associated with melanoma pathogenesis or linked to the response to therapy. The use of the Ion AmpliSeq Cancer Hotspot Panel v2 panel allowed us to detect, starting from as little as 10 ng of DNA, the presence of additional mutations (Figure S1) in the hot spot regions of 50 oncogenes and suppressor genes. Although the clinical utility of targeted NGS in individuals with metastatic melanoma is still debated, the study of unconventional mutations in melanoma samples could become useful in the stratification of patients in the near future [39]. Despite the few cases analyzed in our study, the potentiality deriving from the simultaneous detection of multiple genes results evident. In fact, the identification of additional variants, already reported in databases with a diagnostic or therapeutic meaning (i.e., *KIT, FBXW7, CDKN2A, ERBB4*, and *FLT3*), should be a useful information for the selection of patients in new clinical trials, especially in absence of a classical *BRAF* mutation.

In conclusion, this study demonstrates that targeted-NGS testing is feasible and effective for the routinely accurate detection of mutations in FFPE tissue samples. Although the clinical utility of the NGS technologies needs to be better investigated, in view of new knowledge on molecular markers and the resulting availability of therapeutic targets, the implementation of a flexible and scalable high-throughput system seems to provide a great opportunity for the patient care in the setting of solid tumors as metastatic melanoma.

## Figures and Tables

**Table 1:** Control DNA samples obtained from cell line mixes in different proportions.

			**MIX A**		**MIX B**	**MIX C**
Cell Lines				A375:SW620:HT1197: NCI-H1650	A375:SW620:HT1197:NCI-H1650: NCI-H1975	A375:SW620: HT1197: NCI-H1650: NCI-H1975: SW948
**Proportion in the Mix**				1:1:2:2	1:1:2:2:12	1:1:2:2:12:36
Cell Lines	Mutated Genes	Variant		**Expected VAF (%)**		
A375	BRAF	p.Val600Glu (homozygous)		16.6	5.5	1.8
A375	CDKN2A	p.Glu61Ter (homozygous)		16.6	5.5	1.8
HT1197	NRAS	p.Gln61Arg		16.6	5.5	1.8
HT1197	PIK3CA	p.Glu545Lys		16.6	5.5	1.8
SW620	KRAS	p.Gly12Val (homozygous ^1^)		33.3	11.0	3.6
SW620	SMAD4	c.955 + 5G > C (intronic)		16.6	5.5	1.8
NCI-H1650	APC	p.Ala1358Thr		16.6	5.5	1.8
NCI-H1650	EGFR	p.Glu746_Ala750del		16.6	5.5	1.8
NCI-H1975	PIK3CA	p.Ile391Met			33.3	16.6
NCI-H1975	EGFR	p.Thr790Met			33.3	16.6
NCI-H1975	EGFR	p.Leu858Arg			33.3	16.6
SW948	PIK3CA	p.Glu542Lys				33.3
SW948	APC	p.Arg1114Ter				33.3
SW948	APC	p.Gln1429Ter				33.3
SW948	KRAS	p.Gln61Leu				33.3

Each mutation and expected Variant Allele Frequency (VAF) are reported in relation to the specific dilution mixed sample (A, B and C). ^1^ with a copy gain reported in Berg et al. 2017 [27].

**Table 2:** Results of the analysis performed by Ion Reporter for the expected variants in cell MIX C at the lowest VAF tested.

				Returned Variant Frequency (%)				*p* Value			
Gene	Locus	Change	Exp VAF (%)	C1	C1b	C2	C2b	C 1	C 1b	C2	C 2b
***BRAF***	chr7:140453136	p.Val600Glu	1.8	2.25	1.76	1.77	2.16	0.01338	0.1804	0.22338	0.01236
***CDKN2A***	chr9:21971177	p.Glu61Ter	1.8	1.31 nocall	1.76	REF	REF	0.33961	0.14022		
***NRAS***	chr1:115256529	p.Gln61Arg	1.8	2.24	1.35 nocall	1.55 nocall	2.05	0.00596	0.28338	0.42203	0.02571
***PIK3CA***	chr3:178936091	p.Glu545Lys	1.8	2.06	2.92 *	REF	1.81	0.04845	0.00001 *		0.11086
***SMAD4***	chr18:48586291	c.955 + 5G > C (intronic)	1.8	1.74	1.70	1.82	REF	0.17259	0.1884	0.12453	
***APC***	chr5:112175363	p.Ala1358Thr	1.8	REF	REF	REF	REF				
***EGFR***	chr7:55242464	p.Glu746_Ala750del	1.8	1.53 nocall	1.68	2.20	2.15	0.4187	0.22954	0.00573	0.01061
***KRAS***	chr12:25398284	p.Gly12Val	3.6	4.37 *	4.05 *	5.42 *	4.86 *	0.00001 *	0.00001 *	0.00001 *	0.00001 *

* Low-allele frequency variants called by Ion Reporter with high significant statistical confidence.

## Supplementary Materials

The following are available online at https://www.mdpi.com/2075-4418/9/3/117/s1.
