# Supplementary material for: Analytical Evaluation of an NGS Testing Method for Routine Molecular Diagnostics on Melanoma Formalin-Fixed, Paraffin-Embedded Tumor-Derived DNA
Source: Diagnostics (Basel). 2019 Sep 12;9(3):117. doi: 10.3390/diagnostics9030117 (PMC6787639; doi:10.3390/diagnostics9030117)
Supplement: Supplementary file 1 [file diagnostics-09-00117-s001.pdf]

| Sample with BRAF mutation | NRAS | KRAS | HRAS | CDKN2A | PIK3CA | APC | KIT | ERBB4 | FBXW7 | AKT1 | PDGFRA | TP53 | KDR | EZH2 | FLT3 | None |
|---|---|---|---|---|---|---|---|---|---|---|---|---|---|---|---|---|
| | Gene with additional mutations | | | | | | | | | | | | | | | |
| **Activating BRAF mutations** | | | | | | | | | | | | | | | | |
| MEL2 (p.Val600Glu) | | | | | | | | | | | | | | | | X |
| MEL4 (p.Val600Glu) | | | | p.Arg80Ter | | | | | | | | | | | | |
| MEL6 (p.Val600Glu) | | | | | p.Met1043Ile | | | | | | | | | | | |
| MEL11 (p.Val600Glu complex) | | | | | | p.Thr1292Met | | | | | | | | | | |
| MEL5 (p.Val600Arg) | | | | | | | p.Gly827Glu | p.Asn174Ser | p.Asp567Gly | | | | | | | |
| MEL 12 (p.Val600Lys) | | | | | | | | p.Ser173Phe | | | | | | | | |
| MEL13 (p.Val600_Lys601delinsGlu) | | | | | | | | | | | | | | | | X |
| MEL15-MEL16 (p.Val600Glu at 3.6%) | | | | | | | | | | | | | | | | X |
| MEL21 (p.Val600Arg at 4.9%) | p.Gln61Lys | | | | | | | | | | | | | | | |
| **Intermediate activity BRAF mutations** | | | | | | | | | | | | | | | | |
| MEL7 (p.Asn581Ser) | | | | | p.Cys420Arg | | | | | | | | | | | |
| MEL9 (p.Leu597Gln) | | | | | | p.Glu1317Gln | | | | p.Trp22Leu | | | | | | |
| MEL20-MEL19 (p.Gly466Arg) | | | p.Gly12Asp | p.Arg80Ter | | | | | | | | | | | | |
| **Inactivating BRAF mutations** | | | | | | | | | | | | | | | | |
| MEL8 (p.Asp594Asn) | p.Gly12Asp | | | | | | | | | | | | | | | |
| MEL14 (p.Asp594Asn) | | p.Gln61His | | | | | | | | | | | | | | |
| MEL10 (p.Gly596Val) | p.Gln61Arg | | | | | p.Pro865Ser | | | | | | | | | | |
| **BRAF wild-type** | | | | | | | | | | | | | | | | |
| MEL1 | p.Gln61Leu | | | p.Pro81Leu | | | | | | | p.Gly569Glu | p.Leu257Pro | | | | |
| MEL3 | p.Gln61Arg | | | | | | | | | | | | | | | |
| MEL18-MEL17 | | | | | | | | p.Ser311Phe | | | | | p.Gly1284Arg | p.Tyr646His | p.Trp464Ter | |

ClinVar/SIFT/PolyPhen: Pathogenic or likely pathogenic
ClinVar/SIFT/PolyPhen: conflicting interpretation
ClinVar/SIFT/PolyPhen: benign

**Figure S1. Identified mutations in FFPE samples.** All the identified variants are reported. Each column represents a mutated gene and each row represents a sample. Samples are categorized in relation to the ascribed effect of the mutation on the BRAF protein activity. The additional mutation boxes are highlighted on the basis of the predicted effect.

**Table S1.** Quality parameters and coverage of FFPE samples performed by NGS.

| Sample MEL | Library (pmol/L) | Bases | >=Q20 | Reads | Reads Mean lenght (bp) | Mapped Reads | On Target | Mean Depth | % of amplicon with at least 500x | % of amplicon with at least 100x | Uniformity | Amplicons with lower than 500 reads | Amplicons with lower than 100 reads |
|---|---|---|---|---|---|---|---|---|---|---|---|---|---|
| 1 | 266 | 106728410 | 101938290 | 935415 | 114 | 934005 | 85.46% | 3713 | 98,6 | 100,0 | 98.52% | 3 | 0 |
| 2 | 188 | 132124958 | 126292682 | 1153026 | 115 | 1150097 | 88.83% | 4750 | 99,5 | 100,0 | 94.91% | 1 | 0 |
| 3 | 51 | 110421513 | 105780845 | 983297 | 112 | 958773 | 96.53% | 4344 | 100,0 | 100,0 | 100.00% | 0 | 0 |
| 4 | 150 | 107194613 | 102524590 | 967973 | 111 | 965754 | 87.47% | 3881 | 99,5 | 100,0 | 98.03% | 1 | 0 |
| 5 | 186 | 112826451 | 107891647 | 1007614 | 112 | 1005357 | 84.76% | 3965 | 99,0 | 100,0 | 98.52% | 2 | 0 |
| 6 | 311 | 118383119 | 112991229 | 999407 | 118 | 998243 | 88.16% | 4156 | 99,5 | 100,0 | 98.17% | 1 | 0 |
| 7 | 187 | 114968508 | 109009271 | 1022823 | 112 | 1020411 | 97.01% | 4543 | 95,7 | 100,0 | 89.00% | 9 | 0 |
| 8 | 90 | 65428547 | 61979152 | 608819 | 107 | 607085 | 93.48% | 2553 | 94,7 | 100,0 | 92.75% | 11 | 0 |
| 9 | 75 | 55847911 | 52996111 | 572171 | 98 | 570357 | 82.08% | 2006 | 94,7 | 100,0 | 96.29% | 11 | 0 |
| 10 | 662 | 114570811 | 108203065 | 979578 | 117 | 978676 | 95.54% | 4426 | 99,0 | 100,0 | 98.22% | 2 | 0 |
| 11 | 128 | 75338077 | 71497953 | 687284 | 110 | 686401 | 96.68% | 2992 | 98,1 | 100,0 | 96.77% | 4 | 0 |
| 12 | 386 | 92883182 | 88223655 | 812907 | 114 | 812294 | 97.38% | 3693 | 99,5 | 100,0 | 98.08% | 1 | 0 |
| 13 | 1169 | 105638869 | 100157652 | 904544 | 117 | 903833 | 97.04% | 4101 | 99,0 | 99,5 | 97.95% | 2 | 1 |
| 14 | 412 | 128754379 | 122066099 | 1134610 | 113 | 1133565 | 96.75% | 5086 | 99,5 | 100,0 | 98.46% | 1 | 0 |
| 15 | 99 | 50348751 | 47598296 | 467207 | 108 | 466411 | 92.21% | 1919 | 86,5 | 99,0 | 87.68% | 28 | 2 |
| 16 | 492 | 73629701 | 69815499 | 643732 | 114 | 643152 | 93.58% | 2795 | 97,6 | 99,5 | 96.79% | 5 | 1 |
| 17 | 376 | 72330552 | 68350119 | 629290 | 115 | 628600 | 96.12% | 2837 | 98,6 | 100,0 | 96.46% | 3 | 0 |
| 18 | 132 | 54799548 | 51938524 | 505571 | 108 | 505091 | 97.83% | 2227 | 97,1 | 100,0 | 96.68% | 6 | 0 |
| 19 | 117 | 53572368 | 50636789 | 485481 | 110 | 484838 | 94.30% | 2098 | 97,1 | 100,0 | 97.59% | 6 | 0 |
| 20 | 34 | 38650584 | 36522475 | 363853 | 106 | 362584 | 96.41% | 1545 | 78,3 | 97,6 | 87.86% | 45 | 5 |
| 21 | 228 | 49452569 | 46956005 | 456092 | 108 | 455708 | 97.20% | 2002 | 98,1 | 100,0 | 98.27% | 4 | 0 |

**Table S2.** Analysis of samples with classical BRAF or NRAS variants: comparison of results among different methods.

| | | **Conventional methods** | | |
|---|---|---|---|---|
| **SAMPLE** | **NGS (mutant allele fraction)** | **Sequenom** | **HRMA+Sanger** | **castPCR for *BRAF* p.Val600Glu (mutant allele fraction)** |
| **MEL-1** | BRAF wt | BRAF wt | BRAF wt | BRAF wt |
| | NRAS p.Gln61Leu (40.1%) | NRAS p.Gln61Leu | not performed | not performed |
| | KRAS wt | KRAS wt | not performed | not performed |
| | PIK3CA wt | PIK3CA wt | not performed | not performed |
| **MEL-2** | BRAF p.Val600Glu (47.7%) | BRAF p.Val600Glu | not performed | not performed |
| | NRAS wt | NRAS wt | not performed | not performed |
| | KRAS wt | KRAS wt | not performed | not performed |
| | PIK3CA wt | PIK3CA wt | not performed | not performed |
| **MEL-3** | BRAF wt | BRAF wt | BRAF wt | BRAF wt |
| | NRAS p.Gln61Arg (8.4%) | NRAS p.Gln61Arg | not performed | not performed |
| | KRAS wt | KRAS wt | not performed | not performed |
| | PIK3CA wt | PIK3CA wt | not performed | not performed |
| **MEL-4** | BRAF p.Val600Glu (31.3%) | BRAF p.Val600Glu | not performed | not performed |
| | NRAS wt | NRAS wt | not performed | not performed |
| | KRAS wt | KRAS wt | not performed | not performed |
| | PIK3CA wt | PIK3CA wt | not performed | not performed |
| **MEL-5** | BRAF p.Val600Arg (23.8%) | BRAF p.Val600Arg | not performed | not performed |

| | | | | |
|---|---|---|---|---|
| | NRAS wt | NRAS wt | not performed | not performed |
| | KRAS wt | KRAS wt | not performed | not performed |
| | PIK3CA wt | PIK3CA wt | not performed | not performed |
| **MEL-6** | BRAF p.Val600Glu (46.1%) | BRAF p.Val600Glu | not performed | not performed |
| | NRAS wt | NRAS wt | not performed | not performed |
| | KRAS wt | KRAS wt | not performed | not performed |
| | PIK3CA p.Met1043Ile (44.2%); p.Ile391Met (63.9) | PIK3CA p.Met1043Ile; wt* | not performed | not performed |
| **MEL-11** | BRAF p.Val600Glu complex (38.1%) | BRAF p.Val600Glu complex/p.Val600Asp | not performed | not performed |
| | NRAS wt | NRAS wt | not performed | not performed |
| | KRAS wt | KRAS wt | not performed | not performed |
| | PIK3CA wt | PIK3CA wt | not performed | not performed |
| **MEL-12** | BRAF p.Val600Lys (60.9%) | BRAF p.Val600Lys | not performed | not performed |
| | NRAS wt | NRAS wt | not performed | not performed |
| | KRAS wt | KRAS wt | not performed | not performed |
| | PIK3CA wt | PIK3CA wt | not performed | not performed |
| **MEL-13** | BRAF p.Val600_Lys601delinsGlu (44.0%) | BRAF p.Val600_Lys601delinsGlu | not performed | not performed |
| | NRAS wt | NRAS wt | not performed | not performed |
| | KRAS wt | KRAS wt | not performed | not performed |
| | PIK3CA wt | PIK3CA wt | not performed | not performed |

***mutation not covered by the panel**

**Table S3.** Analysis of samples with BRAF mutated allele at low frequency: comparison of results among different methods.

| | | Conventional methods | | |
|---|---|---|---|---|
| **SAMPLE** | **NGS (mutant allele fraction)** | **Sequenom** | **HRMA+Sanger** | **castPCR for *BRAF* p.Val600Glu (mutant allele fraction)** |
| **MEL-15** | BRAF p.Val600Glu (3.62%) | BRAF wt | BRAF wt | BRAF p.Val600Glu (1.49%) |
| | NRAS wt | NRAS wt | not performed | not performed |
| | KRAS wt | KRAS wt | not performed | not performed |
| | PIK3CA wt | PIK3CA wt | not performed | not performed |
| **MEL-21** | BRAF p.Val600Arg (4.9%) | BRAF wt | BRAF **ambigous** | BRAF p.Val600Arg (8.99%) |
| | NRAS p.Gln61Lys (19.5%) | NRAS wt | NRAS p.Gln61Lys | not performed |
| | KRAS wt | KRAS wt | not performed | not performed |
| | PIK3CA wt | PIK3CA wt | not performed | not performed |

**Table S4.** Analysis of samples with a rarer *BRAF* mutation: comparison of results among different methods.

| | | Conventional methods | | |
|---|---|---|---|---|
| **SAMPLE** | **NGS (mutant allele fraction)** | **Sequenom** | **HRMA+Sanger** | **castPCR for *BRAF* p.Val600Glu (mutant allele fraction)** |
| **MEL-7** | BRAF p.Asn581Ser (44.2%) | BRAF wt * | BRAF p.Asn581Ser | not performed |
| | NRAS wt | NRAS wt | not performed | not performed |
| | KRAS wt | KRAS wt | not performed | not performed |
| | PIK3CA p.Cys420Arg (13.9%) | PIK3CA p.Cys420Arg | not performed | not performed |

| | | | | |
|---|---|---|---|---|
| **MEL-8** | BRAF p.Asp594Asn (52.1%) | BRAF wt * | BRAF p.Asp594Asn | not performed |
| | NRAS p.Gly12Asp (63.4%) | NRAS p.Gly12Asp | NRAS p.Gly12Asp | not performed |
| | KRAS wt | KRAS wt | not performed | not performed |
| | PIK3CA wt | PIK3CA wt | not performed | not performed |
| **MEL-9** | BRAF p.Leu597Gln (25.3%) | BRAF wt * | BRAF p.Leu597Gln | not performed |
| | NRAS wt | NRAS wt | not performed | not performed |
| | KRAS wt | KRAS wt | not performed | not performed |
| | PIK3CA wt | PIK3CA wt | not performed | not performed |
| **MEL-10** | BRAF p.Gly596Val (52.6%) | BRAF wt * | BRAF p.Gly596Val | not performed |
| | NRAS wt | NRAS wt | not performed | not performed |
| | KRAS wt | KRAS wt | not performed | not performed |
| | PIK3CA wt | PIK3CA wt | not performed | not performed |
| **MEL-14** | BRAF p.Asp594Asn (36.8%) | BRAF wt * | BRAF p.Asp594Asn | not performed |
| | NRAS wt | NRAS wt | not performed | not performed |
| | KRAS p.Gln61His (61.2%) | KRAS p.Gln61His | KRAS p.Gln61His | not performed |
| | PIK3CA wt | PIK3CA wt | not performed | not performed |

***mutation not covered by the panel**

**Table S5.** Analysis of wild type samples both for *BRAF* and *NRAS* genes by conventional methods: comparison of results with NGS analysis.

| | | Conventional methods | | |
|---|---|---|---|---|
| **SAMPLE** | **NGS (mutant allele fraction)** | **Sequenom** | **HRMA+Sanger** | **castPCR for *BRAF* p.Val600Glu (mutant allele fraction)** |
| **MEL-16** | BRAF wt | BRAF wt | BRAF wt | BRAF wt |
| | NRAS wt | NRAS wt | not performed | not performed |
| | KRAS wt | KRAS wt | not performed | not performed |
| | PIK3CA wt | PIK3CA wt | not performed | not performed |
| **MEL-17** | BRAF wt | BRAF wt | BRAF wt | BRAF wt |
| | NRAS wt | NRAS wt | not performed | not performed |
| | KRAS wt | KRAS wt | not performed | not performed |
| | PIK3CA wt | PIK3CA wt | not performed | not performed |
| **MEL-18** | BRAF wt | BRAF wt | BRAF wt | BRAF wt |
| | NRAS wt | NRAS wt | not performed | not performed |
| | KRAS wt | KRAS wt | not performed | not performed |
| | PIK3CA wt | PIK3CA wt | not performed | not performed |
| **MEL-19** | BRAF p.Gly466Arg in exon11 (36.0%) | BRAF wt* | BRAF wt | BRAF wt |
| | NRAS wt | NRAS wt | not performed | not performed |
| | KRAS wt | KRAS wt | not performed | not performed |
| | PIK3CA wt | PIK3CA wt | not performed | not performed |
| **MEL-20** | BRAF p.Gly466Arg in exon11 (31.5%) | BRAF wt* | BRAF wt | BRAF wt |
| | NRAS wt | NRAS wt | not performed | not performed |
| | KRAS wt | KRAS wt | not performed | not performed |
| | PIK3CA wt | PIK3CA wt | not performed | not performed |

***mutation not covered by the panel**
